# Pacing the Impossible: ICE‐ and Voltage‐Guided Atrial Leadless Pacemaker Implantation in Double Mechanical Valve Disease

**DOI:** 10.1002/joa3.70405

**Published:** 2026-07-02

**Authors:** Kridhitach Ngarmukos, Narut Prasitlumkum, Pattara Rattanawong

**Affiliations:** ^1^ Department of Medicine, John A. Burns School of Medicine University of Hawai’i Honolulu Hawaii USA; ^2^ Sport and Genetic Electrophysiology Clinic, Pali Momi Heart Center, Pali Momi Medical Center Hawaii Pacific Health Honolulu Hawaii USA

**Keywords:** electroanatomic mapping, intracardiac echocardiography, leadless pacemaker, sinus node dysfunction, tricuspid valve prosthesis

## Abstract

A and B: Right atrial voltage mapping identified a limited region of viable myocardium within an extensive atrial scar. C: Intracardiac echocardiography guided safe deployment of an atrial leadless pacemaker. D and E: Fluoroscopy confirmed stable device positioning in the right atrial appendage of the patient.

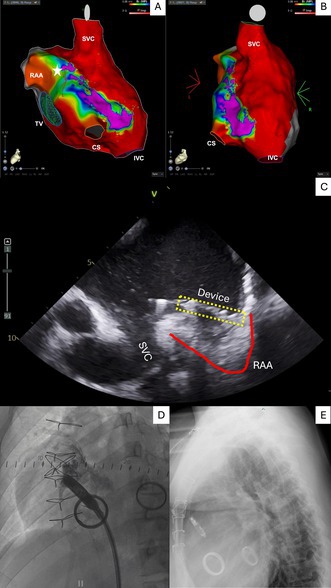

A 60‐year‐old woman with a history of rheumatic heart disease, mechanical mitral and tricuspid valve replacement, multiple catheter ablation procedures for atrial tachyarrhythmias, and prior bi‐atrial Maze surgery presented with recurrent presyncope and near‐syncope. Ambulatory monitoring demonstrated paroxysmal atrial fibrillation with conversion pauses of up to 6 s, consistent with symptomatic sinus node dysfunction. Baseline junctional bradycardia further limited the use of additional rate‐controlling or antiarrhythmic therapy. The patient was maintained on chronic warfarin therapy. Transthoracic echocardiography demonstrated preserved left ventricular systolic function and normally functioning prosthetic mitral and tricuspid valves, without evidence of structural abnormalities that would otherwise explain her symptoms. Given the recurrent symptomatic pauses and inability to optimize medical therapy, permanent pacing was indicated.

Transvenous right ventricular pacing was contraindicated because of the mechanical tricuspid valve. Coronary sinus venography demonstrated occlusion, preventing left ventricular pacing. Surgical epicardial pacemaker implantation was considered but deemed high risk. Although transvenous atrial pacing was theoretically possible, concerns were raised regarding device‐related infective endocarditis in the presence of prosthetic valves and the increased risk of pocket hematoma associated with uninterrupted anticoagulation.

Given that the primary indication was sinus node dysfunction without a need for ventricular pacing, atrial leadless pacing was considered as an alternative strategy. This approach offered the potential advantages of avoiding transvenous leads and generator pockets while providing atrial support to prevent prolonged conversion pauses.

Review of prior high‐density right atrial voltage mapping obtained 10 months earlier during catheter ablation demonstrated diffuse atrial scarring, with a discrete region of preserved voltage localized to the anterior base of the right atrial appendage (Figure 1). This region represented the only viable atrial myocardium suitable for stable pacing and was identified as the optimal target for atrial leadless pacemaker deployment. Repeat mapping was deferred because comprehensive electroanatomic data were already available and because additional mapping could have prolonged the procedure, increased anesthesia exposure, and heightened the risk of catheter entrapment within the mechanical tricuspid valve.

**FIGURE 1:**
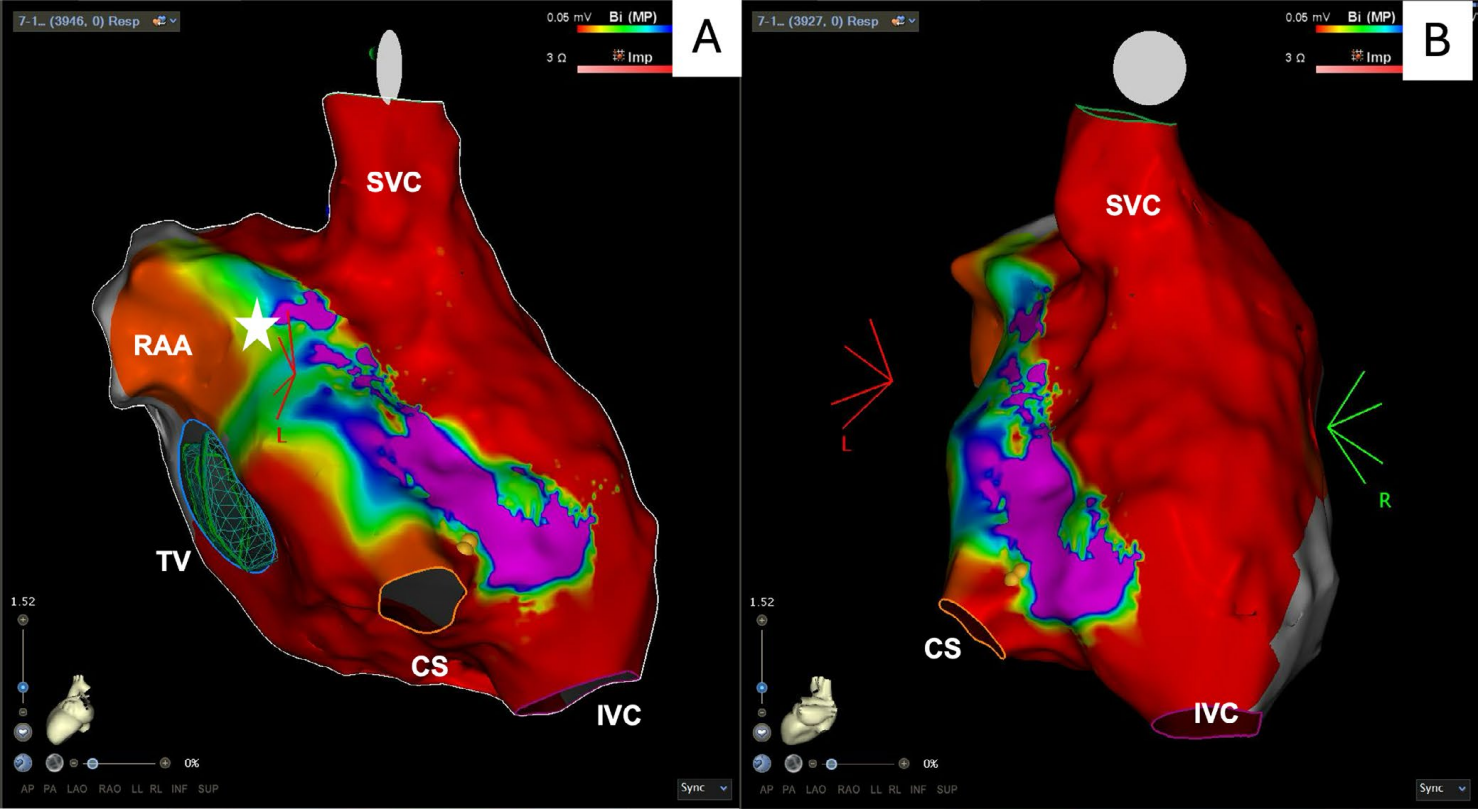
Left lateral (A) and posterior‐anterior (B) voltage map of the right atrium demonstrating areas of scar (red) and preserved viable myocardium (nonred), with target implantation site indicated by the star (A).

The procedure was performed via femoral venous access under general anesthesia. ICE was used to continuously guide catheter navigation and device deployment within the right atrial appendage (Figure 2A and Video S1). An atrial leadless pacemaker was advanced into the right atrium and positioned at the voltage‐defined target site. Perpendicular contact with viable atrial myocardium was confirmed by ICE before deployment. Following release, device stability was verified fluoroscopically (Figure 2B and Video S2). Acute electrical testing demonstrated an atrial capture threshold of 4.0 V at 1.0 ms prior to deployment, which improved to 3.0 V at 1.0 ms immediately after deployment and stabilized at 2.25 V at 1.0 ms following device release. Successful implantation was achieved on the first deployment without the need for device recapture or repositioning. Adequate sensing and normal impedance were maintained throughout the procedure and on the next day, prior to discharge. The device was programmed in AAI mode at a lower rate limit of 40 beats/min. No procedural complications occurred, including pericardial effusion, device dislodgement, or interference with the mechanical tricuspid valve. At discharge, device interrogation demonstrated a stable atrial capture threshold of 2.25 V at 1.0 ms, and postprocedural chest radiography confirmed stable device position (Figure 2C).

**FIGURE 2:**
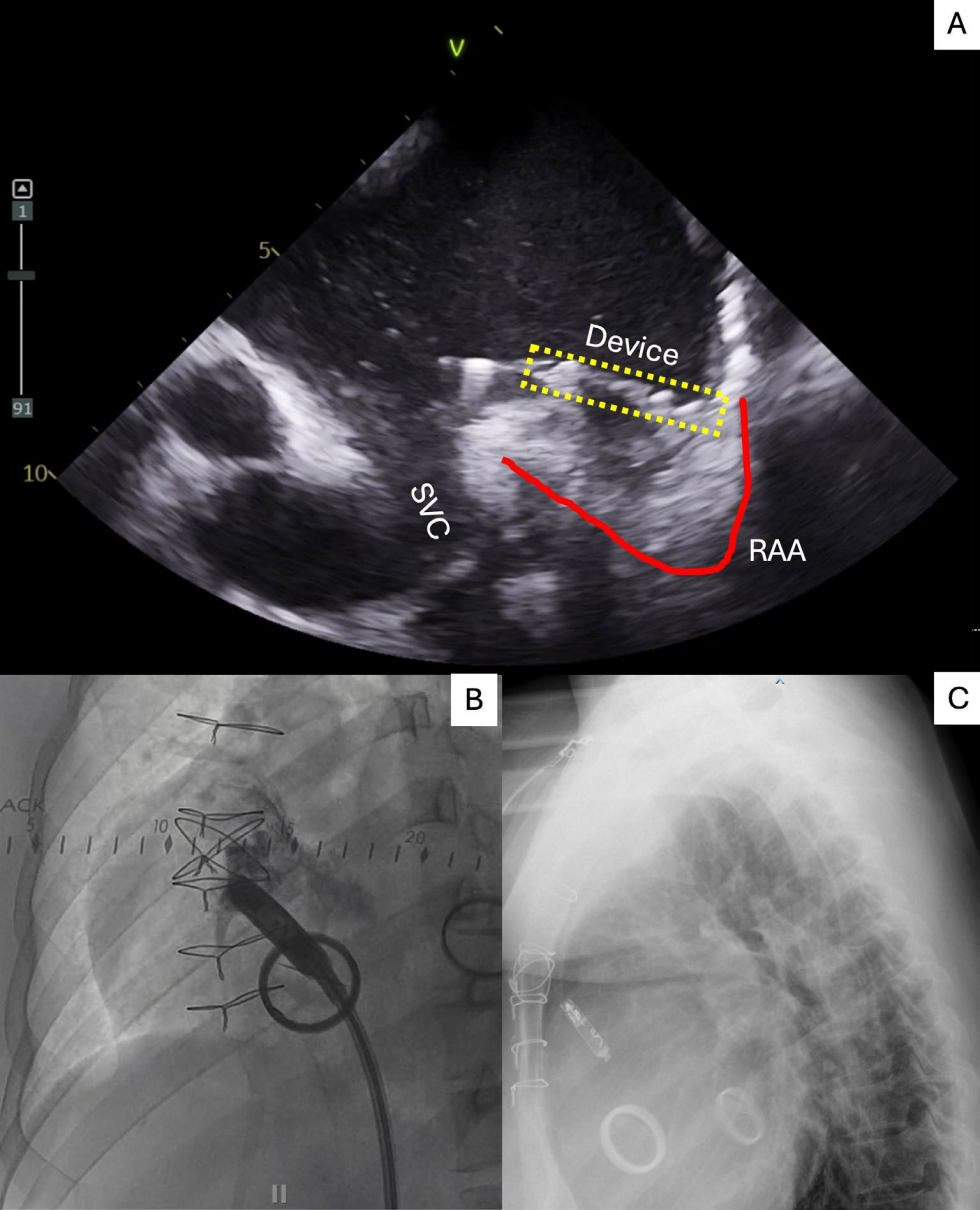
Intracardiac echocardiography (A), fluoroscopy (B), and chest radiography (C) confirming device positioning.

Following implantation, antiarrhythmic therapy with dofetilide and metoprolol was initiated safely, resulting in improved rhythm control. At one‐month follow‐up, device interrogation demonstrated stable sensing with an atrial capture threshold of 2.25 V at 1.0 ms. The atrial pacing burden was 6%, and projected battery longevity was approximately 11.5 years despite the relatively elevated threshold because of the low pacing burden. At seven‐month follow‐up, the atrial capture threshold improved to 1.5 V at 1.0 ms. The atrial pacing burden was 2%, projected battery longevity was approximately 10.8 years, and the atrial tachyarrhythmia burden was 5%. Importantly, the patient reported complete resolution of presyncopal symptoms, with no recurrent presyncope, near‐syncope, or syncope. No sensing abnormalities, device‐related complications, or interactions with the mechanical tricuspid valve were observed during follow‐up.

Patients with mechanical tricuspid valves represent a uniquely challenging population for permanent pacing. Traditional alternatives, such as surgical epicardial pacing, are associated with higher procedural risk [1], while coronary sinus pacing may be anatomically infeasible. Leadless pacing systems offer an attractive alternative by avoiding transvenous leads and subcutaneous pockets, thereby reducing the risks of infection [2] and pocket‐related complications such as hematomas, particularly in patients who are anticoagulated during implantation [3].

Atrial leadless pacemaker implantation is technically demanding, especially in patients with extensive atrial scarring from prior surgery or ablation. In this case, prior voltage mapping was critical in identifying viable myocardium capable of supporting stable atrial pacing [4]. Without electroanatomic guidance, device deployment into heavily scarred atrial tissue could have resulted in poor sensing, elevated capture thresholds, and multiple deployment attempts. Electrical parameters, including an impedance rise and the presence of an injury current, served as the primary indicators of adequate myocardial contact. ICE provided complementary real‐time anatomic guidance following the initial contrast injection, allowing assessment of device position and contact without additional contrast administration [5]. In this complex case involving a mechanical tricuspid valve and a limited area of viable atrial myocardium, ICE served as a useful adjunctive tool to support first‐pass deployment and minimize the need for recapture or redeployment. Together, these complementary modalities enabled accurate targeting of viable atrial myocardium while enhancing procedural safety.

Although ventricular leadless pacing is now well established, experience with atrial leadless systems remains limited. This case illustrates that atrial leadless pacing can be successfully performed even in complex postsurgical anatomy when guided by detailed electroanatomic and imaging information.

Importantly, this strategy allowed avoidance of repeat sternotomy and transvenous hardware in a patient at elevated risk for surgical and infectious complications. While atrial‐only pacing is not appropriate for all patients, it may represent a valuable option for individuals with sinus node dysfunction who lack feasible ventricular pacing access.

In patients with double (tricuspid and mitral) mechanical valves and no viable right or left ventricular pacing options, atrial leadless pacemaker implantation guided by prior voltage mapping with adjunctive intracardiac echocardiography may provide a safe, feasible, and less invasive alternative to surgical epicardial pacing.

## Disclosure

The authors have nothing to report.

## Ethics Statement

Ethical approval was not required for this study in accordance with local institutional policies, as this is a single case report using fully anonymous patient data.

## Consent

Informed consent was obtained from the patient for publication of this case report and any images.

## Conflicts of Interest

The authors declare no conflicts of interest.

## Supporting information


**Video S1:** ICE‐guided deployment of an atrial leadless pacemaker within the right atrial appendage. ICE was used to visualize catheter positioning, confirm perpendicular contact with viable atrial myocardium at the voltage‐guided target site, and guide device deployment in real time.

**Video S2:** Fluoroscopic visualization of atrial leadless pacemaker positioning within the right atrial appendage following contrast injection. Contrast angiography delineates appendage anatomy and facilitates accurate device placement before deployment.

## Data Availability

Data sharing not applicable to this article as no datasets were generated or analysed during the current study.
