# Correction to “Pacing the Impossible: ICE‐ and Voltage‐Guided Atrial Leadless Pacemaker Implantation in Double Mechanical Valve Disease”

**DOI:** 10.1002/joa3.70453

**Published:** 2026-09-15

K. Ngarmukos , N. Prasitlumkum , P. Rattanawong , “Pacing the Impossible: ICE‐ and Voltage‐Guided Atrial Leadless Pacemaker Implantation in Double Mechanical Valve Disease,” Journal of Arrhythmia 42, no. 4 (2026): e70405, 10.1002/joa3.70405.42394817 PMC13324807

We recently noticed a typographical error regarding the patient's age in the first sentence of our published manuscript. The phrase “A 60‐year‐old woman” should be corrected to “A 46‐year‐old woman.”

We apologize for this error.

